# Prevalence of Depression and Anxiety in Patients With Newly Diagnosed Axial Spondyloarthritis: A Cross-Sectional Study

**DOI:** 10.7759/cureus.111716

**Published:** 2026-06-29

**Authors:** Niromie Sridaran, Thomas Cox, Sian Bamford, Luke Kostanjsek, Jack Shi Jie Yuan-Doré, Syed-Ali Tahir, Lara Askari-Ugarte, Jolyon Poole, Hasan Tahir

**Affiliations:** 1 Department of Emergency Medicine, Gold Coast University Hospital, Queensland, AUS; 2 Department of Emergency Medicine, Royal Free London NHS Foundation Trust, London, GBR; 3 Department of Physiotherapy, Royal Free London NHS Foundation Trust, London, GBR; 4 Department of Acute Medicine, Royal Free London NHS Foundation Trust, London, GBR; 5 Department of Rheumatology, Royal Free London NHS Foundation Trust, London, GBR; 6 School of Medicine, Imperial College London, London, GBR; 7 Department of Pain Management, Royal Free London NHS Foundation Trust, London, GBR; 8 Division of Medicine, University College London, London, GBR

**Keywords:** anxiety, axial spondyloarthritis, depression, gad-7 (generalized anxiety disorder-7 item scale), patient health questionnaire-9 (phq-9), prevalence, prevalence‎, rheumatology

## Abstract

Introduction: While the musculoskeletal symptoms of axial spondyloarthritis (axSpA) are well appreciated, emerging evidence demonstrates that patients newly diagnosed with axSpA experience a substantial mental health burden. There is increasing awareness of the need for mental health screening and integrated psychological support as part of comprehensive axSpA management. The primary aims of our study were to assess the prevalence of depression and anxiety in patients with a new diagnosis of axSpA within the past 24 months and to establish the relationship between axSpA disease activity and the severity of anxiety or depression symptoms. The secondary exploratory analysis was to assess the relationship between interventions received (none, biologics, physiotherapy, and patient support groups) and the severity of anxiety or depression symptoms.

Methods: We conducted a single-centre cross-sectional observational study in a tertiary axSpA rheumatology clinic. Of the 95 eligible patients, 66 patients (response rate 69.5%) completed a comprehensive questionnaire assessing patient demographics, disease activity using the Bath Ankylosing Spondylitis Disease Activity Index (BASDAI), quality of life using the Ankylosing Spondylitis Quality of Life (ASQoL) score, and depressive and anxiety symptoms using standardised screening tools, Patient Health Questionnaire-9 (PHQ-9) and Generalised Anxiety Disorder-7 (GAD-7), respectively. Linear regression analysis was performed to assess the correlation between BASDAI and PHQ-9, as well as GAD-7.

Results: Based on the PHQ-9 scores, 81.8% (54/66) of patients had a score of 5 or more, which is equivalent to having symptoms of mild depression. Of these, 57.4% (n = 31/54) reported moderate-severe symptoms of depression (PHQ-9 ≥10). Similarly, 65.2% (n = 43/66) reported anxiety symptoms, with 53.3% (n = 23/43) experiencing moderate-severe symptoms of anxiety (GAD-7 ≥10). Linear regression analysis demonstrated a strong positive correlation between disease activity (BASDAI) and depression (coefficient = 1.77, 95% CI 1.30-2.23, R² = 0.473, p < 0.0001) and between disease activity and anxiety (coefficient = 1.32, 95% CI 0.79-1.85, R² = 0.279, p < 0.0001). Multivariate analysis demonstrated that disease activity independently predicted both depressive (coefficient 1.79, p value < 0.0001) and anxiety symptoms (coefficient 1.33, p value < 0.0001), even after adjusting for age, gender, employment status, diagnostic delay, and quality of life.

Conclusion: This study demonstrates a high prevalence of undiagnosed depression and anxiety in newly diagnosed axSpA patients, with disease activity as an independent predictor of mental health burden. These findings support the urgent need for routine mental health screening at diagnosis and the integration of psychological care within holistic axSpA management. Further prospective research is required to evaluate optimal screening strategies and psychological intervention models to address this substantial treatment gap.

## Introduction

Axial spondyloarthritis (axSpA) is a chronic inflammatory rheumatic disease mainly affecting the spine and sacroiliac joints. With a global prevalence of 0.1-1.4%, commonly manifesting in early adulthood, it is a systemic disease characterised by pain, stiffness, and fatigue [1]. While the musculoskeletal symptoms of axSpA are well recognised, there is limited research into its impact on mental health in newly diagnosed patients.

Studies at present have suggested that individuals diagnosed with axSpA are at a significantly higher risk of developing mental health conditions, with up to 60.7% of patients at risk [2]. This prevalence substantially exceeds the general population rates of depression (7-8%) and anxiety (3-4%), highlighting a critical burden of psychiatric comorbidity in this population. Chronic pain, fatigue, and functional impairment contribute to psychological distress. This is further exacerbated by delays in diagnosis and uncertainty surrounding the progression of the disease, a significant source of anxiety, thereby increasing disease severity [3]. A meta-analysis by Zhao et al. found that depression was significantly associated with increased disease activity and a poorer quality of life [4]. Their findings support the growing recognition of the need for mental health screening and support services as part of holistic axSpA management.

With existing research indicating a higher prevalence of depression and anxiety in patients with this longstanding disease, the mental health burden in newly diagnosed axSpA patients remains under-explored [2]. This represents a possible clinically significant gap, as early identification of depression and anxiety at the point of diagnosis could enable timely psychological intervention and potentially improve treatment adherence and long-term clinical outcomes.

To the best of our knowledge, this study is the first to investigate the degree of depressive and anxiety symptoms in a newly diagnosed axSpA population, building on the existing literature. The primary aim of this study is to assess the prevalence of depression and anxiety in newly diagnosed patients with axSpA within the past 24 months and to establish the relationship between axSpA disease activity and the severity of anxiety or depression symptoms. The secondary objective of this study was to assess the relationship between interventions received (none, biologics, physiotherapy, and patient support groups) and the severity of anxiety or depression symptoms.

## Materials and methods

Study design and setting

This was a cross-sectional observational study conducted in our tertiary specialist axSpA rheumatology clinic at Barnet and Edgware Community Hospitals within the Royal Free NHS Foundation Trust, London, United Kingdom. The axSpA clinic is a dedicated service within the Rheumatology Department and is comprised of a multidisciplinary team (MDT) including Consultant Rheumatologists, Advanced Practice Physiotherapists, and Rheumatology nurse specialists. This specialist clinic receives referrals for patients where there is a clinical suspicion of axSpA from primary care, with specific referral criteria. Local referral criteria require patients to meet four out of the five criteria: age of onset of back pain less than 45 years old, back pain that is gradual, improves with exercise, does not improve with rest, and pain at night that improves on getting up. This study was carried out between April 2025 and December 2025.

Eligibility and recruitment

Our eligibility criteria for this study involved patients ≥18 years old who were newly diagnosed with axSpA at our tertiary clinic within the past 24 months. All participants involved in this study met the 2009 ASAS (Assessment of SpondyloArthritis International Society) classification criteria and had a clinical diagnosis of axSpA based on assessment of symptoms and interpretation of MRI and/or radiographic imaging by specialist axSpA musculoskeletal radiologists [5]. These patients were identified retrospectively from the Rheumatology axSpA Clinic database at Barnet and Edgware Community Hospitals. While diagnostic data used to confirm eligibility were retrospective in nature, all patient-reported outcomes (Patient Health Questionnaire-9 (PHQ-9), Generalised Anxiety Disorder-7 (GAD-7), Bath Ankylosing Spondylitis Disease Activity Index (BASDAI), and Ankylosing Spondylitis Quality of Life (ASQoL)) were collected prospectively at a single point in time. A total of 95 patients met the eligibility criteria, of whom 66 completed the questionnaire in full for analysis. The response rate was 69.5%.

Data collection

All eligible patients who met the criteria were contacted via email and telephone with a link to complete a comprehensive online survey to assess the following outcomes: demographics (age at diagnosis, gender, ethnicity, and employment status), time to diagnosis from symptom onset, presence of extra-articular manifestations, therapeutic interventions undertaken (biologics, physiotherapy, patient support groups), previous diagnosis of depression and/or anxiety, and completion of current PHQ-9 and GAD-7 questionnaires, an up-to-date BASDAI questionnaire, and an ASQoL questionnaire. PHQ-9 and GAD-7 are validated instruments that are nationally endorsed within UK primary care for mental health screening, demonstrate strong internal consistency and test-retest reliability, and are sensitive to clinical change [6-9]. The PHQ-9 classifies patients as having none/minimal (0-4), mild (5-9), moderate (10-14), moderately severe (15-19), and severe (20-27) depression symptoms. The GAD-7 classifies patients as having mild (5-9), moderate (10-14), and severe (15-21) anxiety symptoms. The survey that we designed, containing all these components, is shown in the Appendices. Unfortunately, as these data were collected through a single combined survey, we were unable to obtain the relevant data for those patients who did not respond to the survey. These data could not be collected from other sources, such as electronic patient records, due to insufficient information being available there. Thus, patients who did not respond were excluded from our analysis.

After each response was received, the questionnaire was initially screened by clinicians for mental health safeguarding concerns. Participants identified as having moderate-to-severe depression (PHQ-9 ≥10) or anxiety (GAD-7 ≥10) were contacted via telephone within 48 hours by a clinician. Their General Practitioner was notified in writing of the screening results, and participants were also signposted to appropriate mental health services. After clinician screening was performed, the data were anonymised for statistical analysis.

Statistical analysis

The primary aim of this statistical analysis was to assess the prevalence of depression and/or anxiety symptoms as measured by PHQ-9 and GAD-7 in this cohort. Following this, univariate and multivariate linear regression analyses were performed to assess the relationship between axSpA disease activity and symptoms of anxiety and depression. Variables included in the multivariate analysis were age, gender, employment status, and time to diagnosis. ASQoL data were not included as part of this analysis because many of the items assessed by the ASQoL overlap with the PHQ-9 and BASDAI questionnaires; hence, they cannot be truly independent variables. Prior to performing linear regression analyses using PHQ-9 and GAD-7 scores, normality of residuals was assessed by the production of QQ plots and the Shapiro-Wilk test. Bonferroni corrections were applied to control for multiple testing. For our other secondary objective, to assess the relationship between interventions received and the severity of anxiety or depression symptoms, this was assessed using two-tailed Student's t-tests, comparing patients who received no interventions, started biologic therapy, received physiotherapy input, and attended patient support groups.

For all regression coefficients, 95% confidence intervals (CIs) were calculated using the standard error and the t-distribution to estimate the precision of the effects. For correlation coefficients, 95% confidence intervals were calculated using a Fisher z-transformation. Statistical significance was defined as a two-sided p-value < 0.05.

The data for this study were extracted, prepared, and analysed using RStudio (Version 2025.09.0, Posit PBC, Boston, Massachusetts).

## Results

Study population

A total of 66 patients were included in our analysis of the 95 eligible patients. All these patients had been diagnosed with axSpA within the last 24 months. The response rate was 69.5% (66/95 patients), and 29 eligible patients did not complete the questionnaire. Regarding the patients who did not respond to the survey, we were unable to collect further information as there were insufficient data within their electronic patient records, and it was not feasible to contact the patients further for these data. Thus, these patients who did not respond were excluded from our analysis, and this will be discussed below as a limitation of this study.

The mean age was 42.6 ± 11.9 years, with 41.8% (n = 28) identifying as male. There was a range of ethnicities, with 54.5% identifying as White British, 13.6% as White Other, and the remaining 31.9% representing other ethnic backgrounds: Asian, Black, and Mixed. The mean duration from symptom onset to diagnosis was 6.14 ± 14.5 years, highlighting a substantial diagnostic delay in this cohort. Table 1 shows further patient demographics, history of illness, and therapeutic intervention details. 

**Table 1:** Population demographics, history of illness and therapeutic interventions. Co-morbidities: heart disease, hypertension, diabetes, hypercholesterolaemia, cancer. Extra-articular manifestations: psoriasis, inflammatory bowel disease, enthesitis, uveitis. BASDAI: Bath Ankylosing Spondyloarthritis Disease Activity Index; ASQoL: Ankylosing Spondyloarthritis Quality of Life.

Characteristics	Value
Demographics	
Age at diagnosis (years), mean ± SD	42.6 ± 11.9
Male, n (%)	28 (41.8%)
Ethnicity: White British	54.5%
Ethnicity: White other	13.6%
Ethnicity: remainder	31.9%
Employed, n (%)	53 (80.3%)
History of Illness	
Time to diagnosis, mean ± SD	6.14 ± 14.5
Presence of extra-articular manifestations + co-morbidities, n (%)	39 (59.1%)
BASDAI score, mean ± SD	6.7 ± 1.1
ASQoL score, mean ± SD	10.6 ± 5.2
Therapeutic interventions	
Biologics, n (%)	37 (56.1%)
Physiotherapy, n (%)	45 (68.2%)
Patient support groups, n (%)	12 (18.2%)

In the total cohort, 27.3% (n = 18/66) of patients self-reported that they had a pre-existing formal diagnosis of depression from a healthcare practitioner. From the PHQ-9 scoring system, 81.8% (n = 54/66) of patients had a score of 5 or more and had symptoms equivalent to mild depression; 57.4% (n = 31/54) reported moderate-severe symptoms of depression (PHQ-9 ≥10). As per Kroenke et al. (2001, a PHQ-9 score ≥10 had a sensitivity of 88% and a specificity of 88% for major depression, therefore suggesting that these patients probably have a high likelihood of undiagnosed depression. Similarly, 31.8% (n = 21/66) of patients self-reported that they had a pre-existing formal diagnosis of anxiety from a healthcare practitioner. However, 65.2% (n = 43/66) reported anxiety symptoms (GAD-7 ≥5), with 53.5% (n = 23/43) reporting moderate-severe symptoms of anxiety (GAD-7 ≥10).

The mean BASDAI score was 6.7 ± 1.1, with 66.7% (n = 44) of patients demonstrating high disease activity (BASDAI ≥4) at the time of the study. More than 50% of patients (59.1%) experienced significant disease burden and impact on quality of life, with a mean ASQoL score of 10.6 ± 5.2. Quality of life was significantly associated with mental health burden: those experiencing a moderate-to-severe impact on quality of life secondary to axSpA symptoms demonstrated higher depressive symptoms (coefficient = 0.696, 95% CI (0.55-0.80), R² = 0.484, p < 0.001) and higher anxiety symptoms (coefficient = 0.598, 95% CI (0.42-0.73), R² = 0.357, p < 0.001).

Disease activity, PHQ-9, and GAD-7 scores

**Figure 1:**
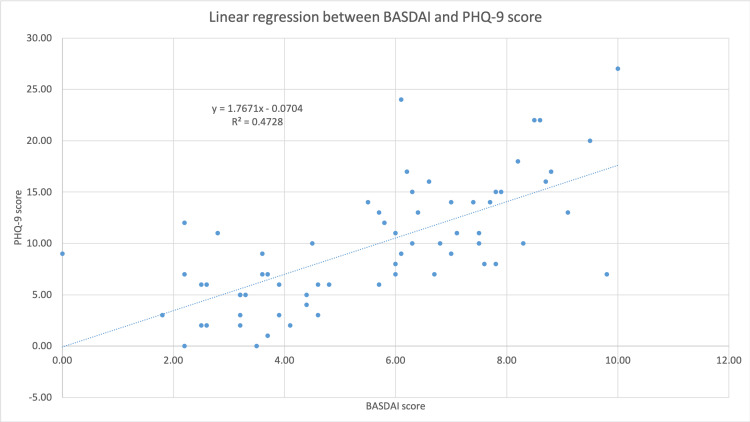
Linear regression analysis showing the relationship between disease activity (BASDAI) and depressive symptoms (PHQ-9). The positive correlation demonstrates that increased disease activity is associated with increased depression scores (coefficient = 1.77, R² = 0.473, p < 0.0001).

**Figure 2:**
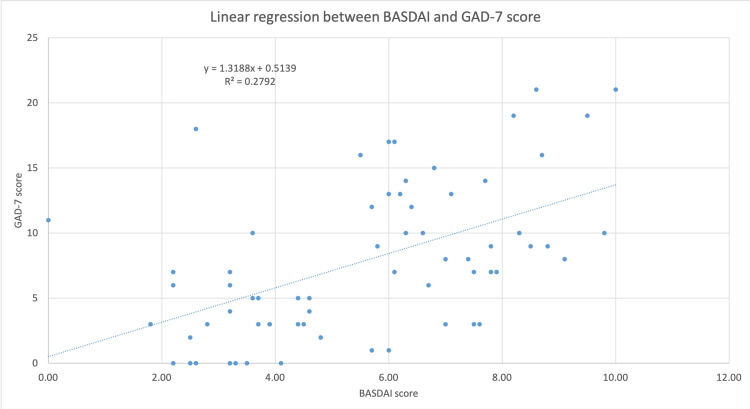
Linear regression analysis showing the relationship between disease activity (BASDAI) and anxiety symptoms (GAD-7). Disease activity accounts for 27.9% of the variance in anxiety scores (coefficient = 1.32, R² = 0.279, p < 0.0001). GAD-7: Generalised Anxiety Disorder-7, BASDAI: Bath Ankylosing Spondylitis Disease Activity Index.

Linear regression analysis was performed to assess the correlation between disease activity (BASDAI) and mental health outcomes (PHQ-9 and GAD-7). There was a strong positive correlation between disease activity and depression (coefficient = 1.77, 95% CI 1.30-2.23, p value < 0.0001), with disease activity accounting for 47.3% of the variability in PHQ-9 scores (R² = 0.473). A similar strong positive correlation was noted between disease activity and anxiety (coefficient = 1.32, 95% CI 0.79-1.85, p value < 0.0001), with disease activity accounting for 27.9% of the variance in GAD-7 scores (R² = 0.279). This correlation is demonstrated in Figures 1, 2. 

Multivariate linear regression analysis was performed to determine whether disease activity was independently associated with mental health burden after adjusting for age, gender, employment status, biologic use, and time to diagnosis. ASQoL data were not included as part of this analysis because many of the items assessed by the ASQoL overlap with the PHQ-9 and BASDAI questionnaires; hence, they cannot be truly independent variables. Ethnicity was not included because, with such a small sample size, many of the reported ethnicities had only a single data point, rendering the analysis vulnerable to imbalance and skewing. This is demonstrated in Tables 2, 3. Even after adjusting for these potential confounders, disease activity (BASDAI) remained a statistically significant independent predictor of depressive symptoms (coefficient = 1.79, 95% CI (1.32-2.27), p < 0.0001) and anxiety symptoms (coefficient = 1.33, 95% CI (0.803-1.85), p < 0.0001). In contrast, age, gender, employment status, and diagnostic delay did not remain statistically significant independent predictors of mental health outcomes in the multivariate model (all p > 0.05). 

**Table 2:** Multivariate analysis of factors that influence the prevalence of depression. A p-value of p < 0.05 was considered statistically significant. BASDAI: Bath Ankylosing Spondylitis Disease Activity Index.

Variable	Co-efficient	95% Confidence Interval	p-value
Age (years)	-0.0304	-0.13 – 0.07	0.55
Gender	0.799	-1.41 – 3.00	0.472
Employment status	0.695	-1.31 – 2.70	0.491
BASDAI	1.79	1.32 – 2.27	< 0.0001
Time to diagnosis (years)	0.000258	-5.31 x 10-10– 0.000569	0.102
Biologics	-0.747	-3.09 – 1.60	0.526

**Table 3:** Multivariate analysis of factors that influence the prevalence of anxiety. A p-value of p < 0.05 was considered statistically significant.

Variable	Co-efficient	95% Confidence Interval	p-value
Age (years)	-0.0292	-0.143 – 0.0843	0.608
Gender	2.54	0.0674 – 5.01	0.0442
Employment status	0.6	-2.82 – 1.65	0.596
BASDAI	1.33	0.803 – 1.858	< 0.0001
Time to diagnosis (years)	0.000186	-0.00016 – 0.000535	0.29
Biologics	-0.135	-2.77 – 2.50	0.919

Treatment and lifestyle interventions

We performed two-sample t-test analysis, as shown in Table 4, assuming equal variances to assess the impact of biologics, physiotherapy, and patient support groups on the prevalence of mental health disease. Patients who received biological therapies and participated in patient support groups had lower mean PHQ-9 scores than those who did not receive these interventions. Similarly, patients who engaged in physiotherapy demonstrated lower mean PHQ-9 and GAD-7 scores compared to those who did not; however, these differences did not reach statistical significance (PHQ-9: t(62) = -0.835, p = 0.407; GAD-7: t(57) = -1.58, p = 0.120). While statistical significance was not achieved, there was evidence of a clinically meaningful trend towards improved mental health outcomes with active therapeutic engagement. The lack of statistical significance is likely attributable to the relatively small sample size and heterogeneous treatment patterns within the cohort. 

**Table 4:** Comparison of depression and anxiety scores between those who engaged with physiotherapy and patient support groups and those who did not. PHQ-9: Patient Health Questionnaire-9, GAD-7: Generalised Anxiety Disorder-7, PSG: patient support groups, df: degrees of freedom.

Two-Sample T-test Analysis	Outcome Measure	
	PHQ-9	GAD-7
Physiotherapy		
Physiotherapy, mean	9.59	8.00
No physiotherapy, mean	10.8	10.4
t (df)	-0.835 (62)	-1.58 (57)
p-value (two-tailed)	0.407	0.120
Patient support groups (PSG)		
PSG mean	9.70	9.55
No PSG mean	10.2	8.24
t (df)	-0.312 (62)	0.890 (57)
p-value (two-tailed)	0.756	0.377

## Discussion

To the authors' knowledge, this is one of the first studies to examine the prevalence of symptoms of depression and anxiety in a newly diagnosed population of axSpA patients, defined as a diagnosis within the last 24 months. The first main finding is that there is a significant prevalence of symptoms of anxiety and depression in patients with a new diagnosis of axSpA in the last 24 months. Our data demonstrate that the prevalence of symptoms equivalent to moderate depression (PHQ-9 ≥10) was 57.4% and symptoms equivalent to moderate anxiety (GAD-7 ≥10) was 53.5%. Of note, these scores of PHQ-9 ≥10 and GAD-7 ≥10 have some diagnostic power, with a previous study by Kroenke et al. demonstrating that they have a sensitivity and specificity of 88% and 88%, respectively [9]. The prevalence of patients with a PHQ-9 ≥10 and GAD-7 ≥10 in our population is consistent with the current literature from Zhao et al. and Reddy et al. [4,10]. However, when compared to the patient self-reported diagnosis of depression and anxiety, which were 27.3% and 31.8%, respectively, this suggests that there may be a discrepancy between reported symptoms of anxiety and depression and receiving a formal diagnosis of depression or anxiety. This adds to the current literature, where Reddy et al. demonstrated the difference between formal diagnoses of depression and anxiety compared to depressive and anxiety symptom burden in axSpA patients. Our data not only continue to highlight that anxiety and depressive symptoms are a problem for axSpA patients but also add to the literature by demonstrating that anxiety and depressive symptoms are significant even in newly diagnosed axSpA patients. Thus, this indicates that mental health symptomatology may be underdiagnosed and therefore potentially under-managed not only in patients with well-established axSpA but also in those with new diagnoses of axSpA.

The second finding from our study is that the severity of these symptoms of depression and anxiety, as measured by PHQ-9 and GAD-7, correlates strongly with disease activity as assessed by BASDAI. This adds to the current literature, with Zhao et al. and Reddy et al. demonstrating similar findings in their studies [4,10]. Notably, our multivariate analysis extends these findings by demonstrating that in newly diagnosed patients, the correlation between disease activity and mental health burden remains statistically significant even after adjusting for age, gender, employment status, and time to diagnosis, suggesting that disease activity is independently associated with depressive and anxiety symptoms. In this analysis, our study demonstrated a mean diagnostic delay of 6.14 years from symptom onset to formal axSpA diagnosis, which is shorter than the mean global time to diagnosis estimated at around 6.7 years [11]. The lack of impact of time to diagnosis on PHQ-9 and GAD-7 scores shown in our data is surprising, especially considering that a systematic review by Yi et al. demonstrated that patients with delayed axSpA diagnosis generally experienced an increased likelihood of depression and higher disease activity [12]. However, this discrepancy between our data and this review may be explained by the differing methodologies of the studies Yi et al. reviewed; none of the studies included in their review utilised PHQ-9 or GAD-7 scores, but instead utilised reported formal diagnoses of depression, which could be underdiagnosed, and the Beck Depression Inventory.

Our study confirms that there is a possible association between mental health and axSpA disease activity; however, given its cross-sectional nature, we are unable to comment on causation. However, we hypothesise that the mechanisms underlying the relationship between disease activity and mental health are likely multifactorial. One possible pathway involves the functional impact hypothesis, in that higher axSpA disease activity impairs physical functioning and increases disability, which directly impacts quality of life and drives depressive and anxiety symptoms. This is supported by our finding that quality of life (ASQoL) was significantly associated with mental health burden (R² = 0.484 for depression, R² = 0.357 for anxiety). A second pathway is supported by the cytokine model of depression, whereby pro-inflammatory cytokines elevated in axSpA, particularly TNF-α and IL-6, directly influence serotonergic and dopaminergic neurotransmission in the central nervous system, thereby contributing to depressive symptoms [13,14]. If this second model is true, it would suggest that more aggressive treatment of axSpA inflammation and earlier initiation of biologic disease-modifying anti-rheumatic drugs (bDMARDs) could possibly provide dual benefits by reducing both musculoskeletal and psychiatric symptoms. However, these hypotheses need further investigation to clarify whether there is a dominant mechanism before specific recommendations can be made.

Study limitations

This study has several limitations inherent to its design as a single-centre cross-sectional study. Firstly, the small overall sample size (n = 66) from a single centre limits generalisability to other UK rheumatology services or international settings, and the findings may not be representative of more diverse populations or healthcare systems with different diagnostic and treatment pathways. The study would merit repeating in different populations. The cross-sectional design provides only a single temporal snapshot of each patient and thus does not permit demonstration of temporal relationships or causality between disease activity and mental health outcomes. Prospective longitudinal data would be required to establish causality more definitively. Finally, the lack of a comparator group, such as healthy controls or other chronic disease cohorts, limits the interpretation of the reported high prevalence rate of anxiety and depressive symptoms in our cohort.

Another major limitation was the 30.5% dropout rate (29/95 patients). As this study utilised patient-reported data from the survey (Appendices), we were unable to obtain the necessary data for those patients who did not complete the questionnaire. Consequently, a formal responder versus non-responder analysis could not be reliably performed. The observed prevalence estimates may have been influenced by non-response and selection bias, as responders may differ from non-responders in terms of mental health burden or disease severity, resulting in an underestimation or overestimation of the results. It is possible that patients experiencing greater psychological distress were more likely to participate, potentially inflating prevalence estimates. Conversely, patients with more severe disease burden or psychological symptoms may have been less likely to engage with questionnaire completion, which could have resulted in an underestimation of the true prevalence. Thus, the high non-response rate may have impacted the validity of our results by potentially introducing sampling bias into our data. This may mean that the data regarding the prevalence of symptoms of depression and anxiety may have been over- or under-represented within our sample population. Similarly, the digital format and questionnaire length may also have introduced digital access bias. Future prospective multi-centre studies should incorporate systematic collection of baseline demographic and clinical data for all eligible participants to enable formal non-responder analyses and more robust assessment of selection bias.

Another limitation of our study is the use of questionnaires and patient self-reported tools. Firstly, while our survey incorporated disease-specific questions alongside validated screening tools (PHQ-9, GAD-7, BASDAI, and ASQoL), these are self-reported measures subject to recall bias. Recall bias would also have affected other outcome measures, including symptom onset and diagnosis dates, which could have been mitigated by cross-referencing with primary care records. Secondly, it is important to note that PHQ-9 and GAD-7 are tools to aid the diagnosis, management, and research of depression and anxiety, and that diagnosis of depression and anxiety is clinically made with reference to the International Classification of Diseases (ICD) or the Diagnostic and Statistical Manual of Mental Disorders (DSM) diagnostic criteria. Thus, the use of PHQ-9 and GAD-7 may potentially over- or underestimate cases depending on threshold definitions, although it is important to note that both these scores have been validated in primary care cohorts with high sensitivity and specificity [6,9,15]. However, these tools have not yet been validated for this specific cohort of axSpA patients, with only two studies to date utilising the PHQ-9, while most other studies have utilised the Hospital Anxiety and Depression Scale [4,10,16]. Another limitation of these tools is that there is significant overlap between the items assessed in the PHQ-9 and GAD-7 and those assessed in BASDAI. One potential route to overcome this issue would have been to use axSpA disease activity measures that incorporate objective measures, such as ASDAS-CRP (Ankylosing Spondylitis Disease Activity Score-C-reactive protein); however, this would not have been feasible due to the design of the data collection using a patient-completed survey. However, despite these limitations with using the PHQ-9 and GAD-7, this does not negate the clinical importance of identifying and treating these symptoms of depression and anxiety, as they are not currently being sufficiently addressed by routine clinical care in axSpA services. This has implications for the management of axSpA, as Reich et al. demonstrated that depressive symptoms have a significant and independent negative effect on patients' response to axSpA treatment, particularly in achieving low disease activity or inactive disease, making the identification and management of co-morbid mental health symptoms crucial from a holistic clinical perspective [17].

Additionally, given that we have performed multiple statistical analyses with our data, this increases the chance of a Type I error. To mitigate this, for our primary outcome, Bonferroni corrections were applied to p-values for the multivariate linear regression analyses, as discussed in our Methods section. However, for the secondary outcomes, no correction was applied as these were performed as exploratory analyses that had been determined pre hoc. Hence, the relationship between interventions and their impact on anxiety and depressive symptoms would need to be formally studied in a longitudinal study with a larger cohort of axSpA patients.

Finally, another issue in our design was that we did not collect information on established risk factors for depression, including family history of depression, marital status, education level, socioeconomic status, and adverse childhood experiences. The absence of these data limits our ability to interpret disease activity as a truly independent predictor of mental health outcomes; unmeasured confounding may explain some of the observed associations. Hence, we would recommend that these metrics be included in future studies examining these psychological symptoms in axSpA patients.

Future directions

Future research should firstly validate the prevalence and incidence of anxiety and depression symptoms in larger multicentre axSpA cohorts using standardised tools and provide prospective longitudinal data on how these might change. The British Axial Spondyloarthritis Cohort (BAxSIC) study, a UK multicentre prospective study examining newly diagnosed axSpA patients, will assess psychological symptomatology alongside disease indices in a prospective framework and will supplement these findings [18].

Secondly, these findings should then be used to develop and validate standardised psychosocial assessment protocols for routine implementation in rheumatology services. Poddubnyy et al. emphasise that, as patients with more severe axSpA symptoms face greater psychological burden, mental health management should form an integral part of axSpA treatment protocols [19]. They recommend developing standardised psychosocial impairment assessment tools to optimise treatment response. These psychological assessment tools should then be incorporated into standard axSpA care models at the time of diagnosis, allowing us to quantify the burden of mental health symptoms and therefore design future axSpA services around this.

Finally, there needs to be further evidence to evaluate the efficacy of specific psychological interventions in reducing depression and anxiety in axSpA populations through randomised controlled trials, as well as to examine whether early treatment of depression and anxiety improves treatment adherence and musculoskeletal outcomes. Current British Society for Rheumatology (BSR) guidelines recommend using a multidisciplinary approach, integrating a psychologist within the MDT, but do not specify the best psychological interventions for axSpA patients [20]. Cognitive behaviour therapy (CBT) has demonstrated efficacy in managing the psychosocial aspects of chronic rheumatological conditions [21,22]. Another angle would be to explore alternative evidence-based approaches, including digital behavioural interventions, as a recent randomised controlled trial demonstrated that digital health applications based on acceptance and commitment therapy reduced emotional distress and may improve health-related quality of life in axSpA patients [23].

## Conclusions

This study demonstrates that there is a high prevalence of depressive and anxiety symptoms in patients newly diagnosed with axSpA, aligning with previous reports in the literature. This represents a clinically significant signal that requires further study, first to evaluate the true prevalence of depressive and anxiety symptoms in different axSpA populations, and secondly to determine the best interventions to manage these symptoms and how this might impact axSpA disease activity. Our data also demonstrate a strong independent correlation between disease activity (BASDAI) and mental health burden, with disease activity accounting for variance in depressive and anxiety symptoms. This relationship persisted after adjusting for age, gender, employment status, and diagnostic delay, suggesting disease activity as a factor independently associated with mental health symptom burden, which may represent a modifiable target pending prospective confirmation.

## Appendices

A table detailing the questionnaire used in this study is as follows. 

**Table 5:** Questionnaire sent to the patients.

SECTION 1 – Patient information					
What is your full name?					
What is your date of birth?					
What is your gender?					Male
					Female
					Non-binary
					Other
					Prefer not to say
What is your ethnicity?					Arab
					Asian (Indian)
					Asian (Pakistani)
					Asian (Bangladeshi)
					Asian (Chinese)
					Asian (other)
					Black (African)
					Black (Caribbean)
					Black (other)
					Mixed (Black and White)
					Mixed (Asian and White)
					Mixed (other)
					White (English, Welsh, Scottish, Northern Irish or British)
					White (Irish)
					White (Gyspy or Irish traveller)
					White (Roma)
					White (other)
					Prefer not to say
					Other
What is your employment status?					Employed
					Unemployed
					Retired
					Student
					Home maker
If you are employed, how many days off work (including sick leave) have you had in the past year due to your Axial Spondyloarthritis condition?					
SECTION 2 – Diagnosis/ Treatment of Axial Spondyloarthritis					
When did you first experience your inflammatory back pain symptoms? (day/ month/ year)					
When were you first diagnosed with Axial Spondyloarthritis? (day/ month/ year)					
Do you have any of these following conditions?					Psoriasis
					Inflammatory Bowel Disease (Ulcerative Colitis, Crohn’s)
					Enthesitis (for example, plantar fasciitis, Achilles tendonitis)
					Uveitis
					Heart disease
					High blood pressure
					Diabetes
					High cholesterol
					Cancer
					None of the above
As part of your treatment, are you receiving any physiotherapy?					Yes
					No
					Have been in the past
As part of your treatment, are you currently on any biologic medication?					Yes
					No
					Have been in the past
How many biologic medications have you been previously treated with?					
Do you engage with any patient support groups for Axial Spondyloarthritis?					Yes
					No
					Sometimes
In what ways does having Axial Spondyloarthritis impact your life?					
SECTION 3 – Mental Health Assessment					
Have you been previously diagnosed with Depression?			Yes		
			No		
If you have answered yes to the above, when were you diagnosed with Depression?			Prior to being diagnosed with Axial Spondyloarthritis		
			After being diagnosed with Axial Spondyloarthritis		
			Not applicable		
Have you been previously diagnosed with Anxiety?			Yes		
			No		
If you have answered yes to the above, when were you diagnosed with Anxiety?			Prior to being diagnosed with Axial Spondyloarthritis		
			After being diagnosed with Axial Spondyloarthritis		
			Not applicable		
How are you coping with the diagnosis of Axial Spondyloarthritis with regards to your mental health?					
What sort of support do you think you need or would benefit from with your Axial Spondyloarthritis?					
In what ways can we support you as a service in managing your Axial Spondyloarthritis?					
Patient Health Questionnaire (PHQ-9) The PHQ-9 is a validated, 9-question tool to assess for the degree of depression present in an individual. Please read each question and select the box you feel is the most appropriate. Please only select one box for each question. Over the last two weeks, how often have you been bothered by any of the following problems?					
Little interest or pleasure in doing things?				Not at all	
				Several days	
				More than half the days	
				Nearly every day	
Feeling down, depressed, or hopeless?				Not at all	
				Several days	
				More than half the days	
				Nearly every day	
Trouble falling or staying asleep, or sleeping too much?				Not at all	
				Several days	
				More than half the days	
				Nearly every day	
Feeling tired or having little energy?				Not at all	
				Several days	
				More than half the days	
				Nearly every day	
Poor appetite or overeating?				Not at all	
				Several days	
				More than half the days	
				Nearly every day	
Feeling bad about yourself - or that you are a failure or have let yourself or your family down?				Not at all	
				Several days	
				More than half the days	
				Nearly every day	
Trouble concentrating on things, such as reading the newspaper or watching television?				Not at all	
				Several days	
				More than half the days	
				Nearly every day	
Moving or speaking so slowly that other people could have noticed? Or the opposite - being so fidgety or restless that you have been moving around a lot more than usual?				Not at all	
				Several days	
				More than half the days	
				Nearly every day	
Thoughts that you would be better off dead, or of hurting yourself in some way?				Not at all	
				Several days	
				More than half the days	
				Nearly every day	
Generalised Anxiety Disorder Questionnaire (GAD-7) The GAD-7 is a validated, 7-question tool to assess for the degree of anxiety present in an individual. Please read each question and select the box you feel is the most appropriate. Please only select one box for each question. Over the last two weeks, how often have you been bothered by any of the following problems?					
Feeling nervous, anxious or on edge?		Not at all			
		Several days			
		More than half the days			
		Nearly every day			
Not being able to stop or control worrying?		Not at all			
		Several days			
		More than half the days			
		Nearly every day			
Worrying too much about different things?		Not at all			
		Several days			
		More than half the days			
		Nearly every day			
Trouble relaxing?		Not at all			
		Several days			
		More than half the days			
		Nearly every day			
Being so restless that it is hard to sit still?		Not at all			
		Several days			
		More than half the days			
		Nearly every day			
Becoming easily annoyed or irritable?		Not at all			
		Several days			
		More than half the days			
		Nearly every day			
Feeling afraid as if something awful might happen?		Not at all			
		Several days			
		More than half the days			
		Nearly every day			
The Bath Axial Spondyloarthritis Disease Activity Index (BASDAI Score) Questionnaire to assess the disease activity in Axial Spondyloarthritis. Please read each question and select the box you feel is the most appropriate to describe how severe your Axial Spondyloarthritis has been in this area. Each question relates to how you have felt in the past week. Please only select one box for each question. There is no wrong answer. Each answer is a score out of 10 (very severe).					
How would you describe the overall level of fatigue/tiredness you have experienced?					
How would you describe the overall level of Axial Spondyloarthritis neck, back or hip pain you have had?					
How would you describe the overall level of pain/swelling in joints other than the neck, back or hips?					
How would you describe the overall level of discomfort you have had from any tender areas to touch or pressure?					
How would you describe the overall level of morning stiffness you have had from the time you wake up?					
How long does your morning stiffness last from the time you wake up? (score 1 = none, score 5 = 1 hour, score 10 = 2 hours or more).					
Ankylosing Spondyloarthritis Quality of Life Questionnaire (ASQoL) Please read each question and select the box you feel is the most appropriate. Please select the answer that applies to you in this moment. Each answer was a yes or no.					
My condition limits the places I can go					
I sometimes feel like crying					
I have difficulty dressing					
I struggle to do jobs around the house					
It’s impossible to sleep					
I am unable to join in activities with my friends/family					
I am tired all the time					
I have to keep stopping what I am doing to rest					
I have unbearable pain					
It takes a long time to get going in the morning					
I am unable to do jobs around the house					
I get tired easily					
I often get frustrated					
The pain is always there					
I feel I miss out on a lot					
I find it difficult to wash my hair					
My condition gets me down					
I worry about letting people down					
Would you like to be sent a summary of the findings from this research project?	Yes				
	No				
